# Age-related differences in embodying a tool under visual deprivation

**DOI:** 10.1007/s00221-026-07382-y

**Published:** 2026-08-28

**Authors:** Amir Jahanian Najafabadi, Bernhard Hommel

**Affiliations:** 1 Department of Cognitive Neuroscience, Bielefeld University, 33501 Bielefeld, Germany; 2 School of Psychology, Shandong Normal University, Jinan, 250061 China

**Keywords:** Tool use, Aging, Visual deprivation, Cane use, Body schema, Reaching distance estimation, Ownership, Agency

## Abstract

Tool embodiment refers to the integration of external objects into action-related body representations. Although vision is often assumed to support this process, it remains unclear how tool use under short-term visual deprivation affects explicit ownership and agency across the adult lifespan. This study examined whether blindfolded cane use elicits subjective ownership and agency in younger and older adults, and whether these experiences are associated with forearm tactile distance perception and perceived reaching distance. Forty-three healthy right-handed adults completed two blindfolded cane-use conditions: object search training and navigation training. Tactile Distance Judgment and Reaching Distance Estimation tasks were administered before and after cane use, and participants rated ownership and agency after each condition. Across age groups and conditions, agency ratings were consistently stronger than ownership ratings. Regression analyses indicated that behavioral recalibration measures, especially Reaching Distance Estimation Error, were more consistently associated with agency than ownership, with the clearest joint-model effect observed for agency during navigation. Age Group also contributed to agency during navigation, although age-dependent interaction effects did not reach statistical significance. These findings suggest that short-term blindfolded cane use can support explicit action-based control over a tool without necessarily producing a strong conscious feeling that the tool belongs to the body. The results highlight a dissociation between explicit ownership, agency, and behavioral indices of tool-related sensorimotor updating under blindfolded tool use.

## Introduction

Tool use provides classical example of body representation plasticity. When humans use external objects to act on the environment, tools can become functionally incorporated into action planning and body-related spatial representations (Cardinali et al. 2009; Miller et al. 2014, 2017a). In the present study, we use the term *tool embodiment* to refer to a broader family of tool-related changes, including explicit feelings of ownership and agency over a tool, as well as indirect behavioral changes in body schema and action-related spatial representation (Jahanian Najafabadi et al. 2026a, b; Longo and Serino 2012; Miller et al. 2014, 2017a). These components should not be treated as identical. Ownership refers to the conscious feeling that an object belongs to, or is part of, the body, whereas agency refers to the feeling of controlling the object’s movements and effects (Kalckert and Ehrsson 2012; Ma and Hommel 2015).

The idea that tools can be incorporated into body-related representations has its historical roots in neurophysiological work by Iriki and colleagues, who showed that tool use can modify body-schema-related coding in macaque parietal neurons (Iriki et al. 1996). In humans, Berti and Frassinetti (2000) provided influential neuropsychological evidence that tool use can remap far space into near/action space, demonstrating that the functional boundaries of body-related spatial representation can be altered by using a tool (Berti and Frassinetti 2000). Subsequent studies extended this evidence by showing that tool use can also modify perceived body metrics. In healthy participants, Sposito and colleagues showed that tool use can increase perceived arm length, providing direct evidence for changes in body representation after tool use (Sposito et al. 2012). Similarly, Garbarini et al. (2015) showed in neurological patients that tool use can modulate pathological embodiment and alter the representation of one’s own body (Garbarini et al. 2015). Together, these findings indicate that tool use can affect both action-related spatial representation and perceived body dimensions, often in the direction of an elongation or extension of the represented limb.

Peripersonal space is also not a unitary construct. It has been conceptualized as reachable space, action space, a multisensory facilitation zone around the body, and defensive space (Brozzoli et al. 2009; Canzoneri et al. 2012; Coello et al. 2008; Graziano and Cooke 2006; Maravita et al. 2002). In the present study, we focus specifically on reachability- or action-related spatial representation termed reaching distance estimation, because participants judged the boundary of what they could reach with the arm. Therefore, the reaching distance estimation task is interpreted as a behavioral proxy for perceived reachability and action-scaled spatial representation, not as a direct measure of defensive or multisensory-facilitation peripersonal space (Coello et al. 2008; Jahanian Najafabadi et al. 2026a, b; Zanini et al. 2021). This distinction is important because reaching space and peripersonal space can dissociate in both spatial extent and multisensory facilitation patterns (Zanini et al. 2021).

Tool use can alter both local body metrics and perceived action boundaries. Tactile distance judgment tasks have been widely used to assess body schema recalibration after tool use, because changes in perceived tactile distance can indicate altered metric representation of the stimulated limb (de Vignemont 2010; De Vignemont et al. 2005; Miller et al. 2014, 2017a, b). In parallel, reachability and distance estimation tasks assess action-scaled spatial representation by asking participants to judge whether objects fall within possible reaching space (Bourgeois et al. 2014; Coello et al. 2008; Witt et al. 2005). These measures are complementary but not equivalent: tactile distance judgment (TDJ) indexes local body-metric recalibration, whereas reaching distance estimation (RDE) indexes explicit perceived reachability while taking the arm length into account.

Studies in younger adults have shown that using tools to interact with distant objects can modify perceived reachability and distance perception (Bahmad et al. 2020; Costello et al. 2015; Martel et al. 2016, 2019; Miller et al. 2014, 2017b). Tool-induced recalibration also appears to be limb-specific and practice-dependent, with changes often observed for the trained effector but not for unrelated body parts (Cardinali et al. 2009, 2021; Miller et al. 2014; Sun and Tang 2019). These findings suggest that active tool use can update action-related body representations, but that the extent of updating depends on the task, body part, and available sensory feedback (Cardinali et al. 2009; Miller et al. 2017a, b).

Vision may play an important role in tool-related recalibration, but its contribution depends on the measure and task context. Some studies suggest that tool use effects on perceived body metrics are reduced or absent when visual feedback is unavailable, indicating that visual spatial attention may facilitate tool-related body schema updating (Martel et al. 2019, 2021). However, short-term visual deprivation can also increase reliance on proprioceptive and tactile information, and may affect proprioceptive recalibration without necessarily producing stronger multisensory ownership (Radziun and Ehrsson 2018). These findings suggest that visual deprivation may influence body schema updating, reachability judgments, ownership, and agency in different ways. Accordingly, the present study treats TDJ, RDE, ownership, and agency as related but separable outcomes.

Agency and ownership may also be shaped by the degree of control over the tool. The ability to control an external object is considered important for integrating it into self-related representations and for generating agency over its actions (Ma and Hommel 2015). Research in virtual reality further shows that sensory feedback and controllability can influence both ownership and agency over artificial or virtual body parts (Kokkinara and Slater 2014; Kong et al. 2017). In our previous virtual tool use studies, visuo-haptic feedback supported stronger practice effects than vision alone, and higher ownership ratings in younger adults were associated with forearm body schema changes measured by TDJ, whereas older adults showed weaker or absent body schema recalibration (Jahanian Najafabadi et al. 2023a, b). These findings suggest that tool embodiment depends not only on tool use itself, but also on the quality of multisensory feedback and the age of the user.

Aging may further modulate tool embodiment because older adults often show changes in sensorimotor precision, proprioceptive reliability, visuospatial attention, and action-scaled spatial judgments (Costello and Bloesch 2017; Sugovic and Witt 2013). Older adults may rely more strongly on visual information during action and spatial judgments, which could reduce the flexibility of tool-related updating when vision is unavailable (Costello and Bloesch 2017). Recent work also suggests that tool use after-effects can be reduced in older adults compared with younger adults, including in studies of action-related spatial updating and peripersonal space modulation (Luyat et al. 2024; O’Leary et al. 2026). At the same time, older adults may still preserve a subjective sense of agency when they actively control a tool, even when explicit ownership over the tool remains weak. This distinction is theoretically important because agency can arise from motor intention and sensorimotor prediction, whereas explicit ownership over a non-corporeal object such as a cane may require stronger multisensory integration (Kalckert and Ehrsson 2012; Ma and Hommel 2015).

Task demands may also influence tool embodiment and sense of control over the tool (Jahanian Najafabadi et al. 2025). Different forms of tool use place different demands on tactile exploration, motor control, body movement, and spatial updating. Object search with a cane requires localized tactile exploration of external space, whereas navigation requires continuous sensorimotor control of the cane during whole-body movement. Because action goals can shape how tools are incorporated into body-related and spatial representations, comparing these two forms of cane use allows us to test whether ownership and agency depend on the functional context in which the tool is used (Cardinali et al. 2009; Miller et al. 2017a; Sun and Tang 2019).

## Current Study

A lifespan study (Jahanian Najafabadi et al. 2026a, b) revealed that in adolescents, higher ownership ratings over a physical tool were associated with perceived reaching distance and forearm tactile distance estimation. However, agency ratings were notably higher in far space (beyond arm’s reach) compared to near space (within arm’s reach), and these ratings were independent of changes in the estimation of reaching distance and forearm tactile distance perception. Among young and mid-age adults, higher ownership ratings were associated with enhanced body schema plasticity and changes in reaching distance perception, particularly in far space compared to near space. Furthermore, young adults demonstrated that higher agency ratings in far space were predicted by changes in the estimation of reaching distance and forearm tactile distance perception. In contrast, older adults did not exhibit a strong sense of ownership over physical tools. Nonetheless, their higher agency ratings were associated with increased body schema and perceived reaching distance estimation, particularly during training in far space highlighting the age-dependency of tool-embodiment and underlying mechanisms.

Building on previous studies in the literature of tool use induced plasticity, visual deprivation, and aging, the present study examined whether blindfolded cane use elicits explicit ownership and agency in younger and older adults, and whether these ratings are associated with perceived forearm tactile distance and perceived reaching distance (Cardinali et al. 2009; Jahanian Najafabadi et al. 2026a, b; Jahanian Najafabadi and Godde 2025; Miller et al. 2014; Radziun and Ehrsson 2018; Zanini et al. 2021). Participants completed two blindfolded cane-use conditions: object search and navigation (Jahanian Najafabadi and Hommel 2025). Object search required localized tactile exploration of external space, whereas navigation required continuous sensorimotor control of the cane during whole-body movement.

We formulated three predictions. First, because participants actively generated cane movements, we expected agency ratings to be stronger than ownership ratings under blindfolded tool use. Second, because navigation requires continuous online control of the cane and body movement through space, we expected agency during navigation to be more strongly associated with perceived forearm tactile distance and perceived reaching distance than agency during object search. Third, because aging is associated with changes in sensorimotor and spatial updating, we expected the association between behavioral recalibration measures and subjective embodiment to be weaker in older adults than in younger adults (Costello and Bloesch 2017; Jahanian Najafabadi et al. 2023b, 2026a, b; Luyat et al. 2024; Sugovic and Witt 2013).

## Method

### Participants

The current study procedure was approved by the ethics committee of Bielefeld University. The younger group consisted of 27 healthy right-handed young adults (Mean_age_ = 23.51; SD = 2.83; 14 female), and the older group of 16 healthy right-handed older adults (Mean_age_ = 63.68; SD = 2.93; 9 female). Participants were recruited from our available pool of participants willing to contribute scientific studies. Participation was voluntary and subjects received compensation. The unbalanced group sizes reflected recruitment feasibility, particularly for the older-adult group. In addition, the blindfolded navigation task required additional safety precautions for older adults, which constrained recruitment and training duration. Participants reported no prior use of cane in any type, no known neurological or neuropsychiatric disorders and were able to understand and complete the task instructions. Therefore, eligibility was based on self-reported health status and task comprehension rather than standardized cognitive assessment. Participants provided informed consent (participation and publication), and were naïve to the experimental hypothesis, acuity and errors.

### Study design and procedure

**Fig. 1:**
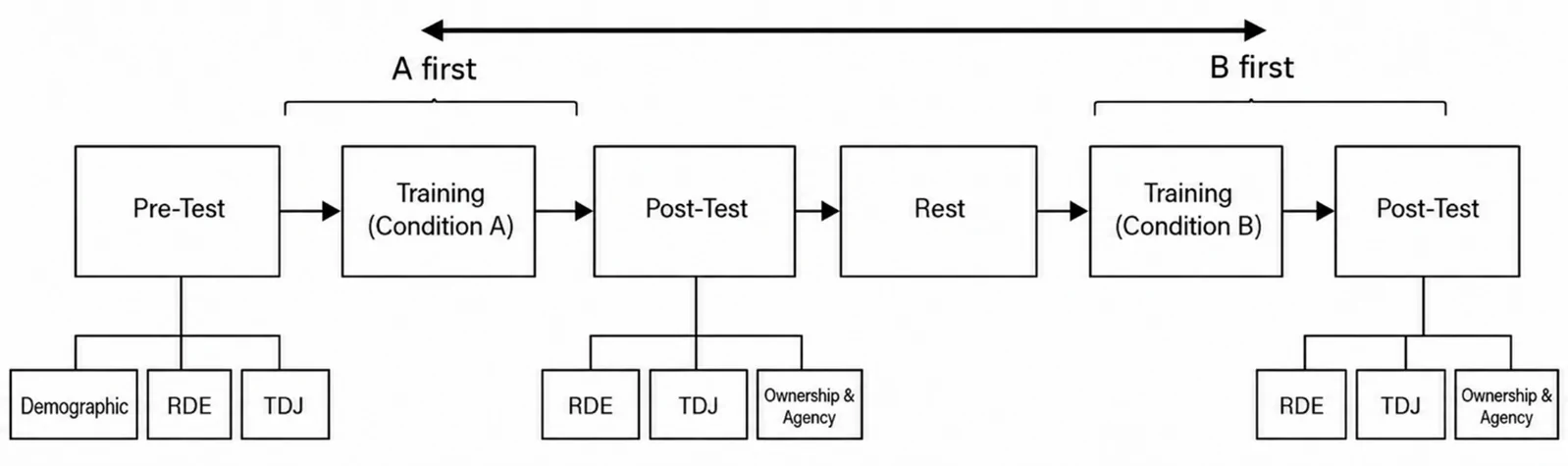
Experimental procedure conducted separately within each age group. Participants completed a pre-test before the first and second training blocks. A post-test was administered after each training block, and a 45-minute rest period separated consecutive blocks. Training conditions were presented in randomized order within each experiment to reduce potential carry-over effects. Abbreviations: Reaching Distance Estimation (RDE), Tactile Distance Judgment (TDJ)

In each age group, participants were measured immediately before and after each of the two training blocks, which are descriptively labeled as Object Search Training (Condition A) and Navigation Training (Condition B). A baseline pre-test was administered prior to the first training block, and an identical post-measurement followed each subsequent block. Between the two blocks, participants were given a 45-minute rest period to eliminate potential carry-over effects. To structurally control for order effects, the presentation sequence of the two training conditions was fully counterbalanced across participants within each age group (cf. Fig. 1). The younger adult cohort was allowed up to 20 min to complete each training block, while the older adult cohort’s training was strictly capped at 10 min per block to ensure safety and prevent motor fatigue.

### Tool use training blocks

The tool use training protocol was adapted from prior research (Sun and Tang 2019), which mirrored their research with distinct functional blocks: Object Search Training and Navigation Training. To prevent potential carry-over and order effects, these blocks were fully counterbalanced across participants within each age group, rather than following a fixed sequence. The entire training session took place under total visual deprivation; participants put on an opaque blindfold from the start of training blocks, which remained on continuously until the start of post-test assessments were complete.

The task paradigms and apparatus were also adapted from prior research (Sun and Tang 2019). While blindfolded, participants manipulated a 120-cm aluminum mobility cane (approx. 300 g) with their dominant right hand. This specific cane was implemented to ensure methodological consistency and a direct baseline replication of their prior work. While a tool of this length is conventionally associated with locomotor navigation, utilizing it for the localized tabletop object search served an important theoretical purpose: it allowed us to keep the structural and mechanical properties of the tool perfectly identical across both experimental conditions. Consequently, any observed variations in forearm tactile distance perception or reaching distance estimation can be attributed purely to the functional shifts in motor dynamics (fine-motor sweeping vs. gross locomotion) rather than physical changes in tool length, weight, or leverage.

To standardize acoustic feedback and reduce environmental sound variability, the cane was equipped with a sound-dampening rubber tip, and participants were instructed to maintain a uniform sweeping technique. The testing room was in size of 6 × 5-meter experimental space completely cleared of peripheral furniture or obstacles, and the experimenter accompanied participants in absolute silence to prevent any directional acoustic cues.

### Object search training

During Object Search Training, participants stood at a fixed distance of 10 cm from the front edge of a customized testing table. The primary motor goal was to explore the tabletop space using the cane to locate a target object via tactile feedback. The target object was a wooden block measuring 4 × 4 × 8 cm. To prevent localization hints, the experimenter placed the object silently on the table in one of 30 predetermined coordinates across 30 sequential trials.

The spatial layout of these coordinates spanned three discrete longitudinal distances from the participant’s body (100 cm, 130 cm, and 160 cm) and ten transverse positions covering a total lateral range of 90 cm. These transverse positions were distributed in 10-cm increments from 45 cm left to 45 cm right of the participant’s sagittal midline. Upon locating the wooden target via sweeping, participants performed a standardized verification sequence by tapping the front, top, left, and right sides of the block with the cane tip. Once the target was verified, the experimenter silently relocated it to the next randomized coordinate.

### Navigation training

The navigation training as Condition B was adapted from the walking-with-cane condition used by Sun and Tang (Sun and Tang 2019). During Navigation Training, participants engaged in gross-motor locomotion guided exclusively by the same 120-cm mobility cane used in Object Search Training. The task was conducted in a cleared 6 × 5 m experimental space with no peripheral furniture or obstacles. Participants began from a fixed starting position and, while blindfolded, used the cane with their dominant right hand to explore the surrounding space and find their way with the help of the tool. They were instructed to use a rhythmic side-to-side sweeping technique, resembling blind cane navigation, to continuously sample the floor area ahead of them and clear a safe walking path. The task did not involve locating a floor target module; rather, the goal was active blindfolded locomotor exploration with the cane. The experimenter accompanied participants silently throughout the task to ensure safety while avoiding directional acoustic cues. The same cane was used in both conditions.

This condition was included to mirror the walking-with-cane condition of Sun and Tang (Sun and Tang 2019), and to test whether tool-related embodiment differs when the same cane is used for gross locomotor navigation rather than localized tabletop object search (Condition A). Thus, the two conditions differed in functional task demands while keeping the physical properties of the tool constant.

### Training duration and safety controls

**Fig. 2:**
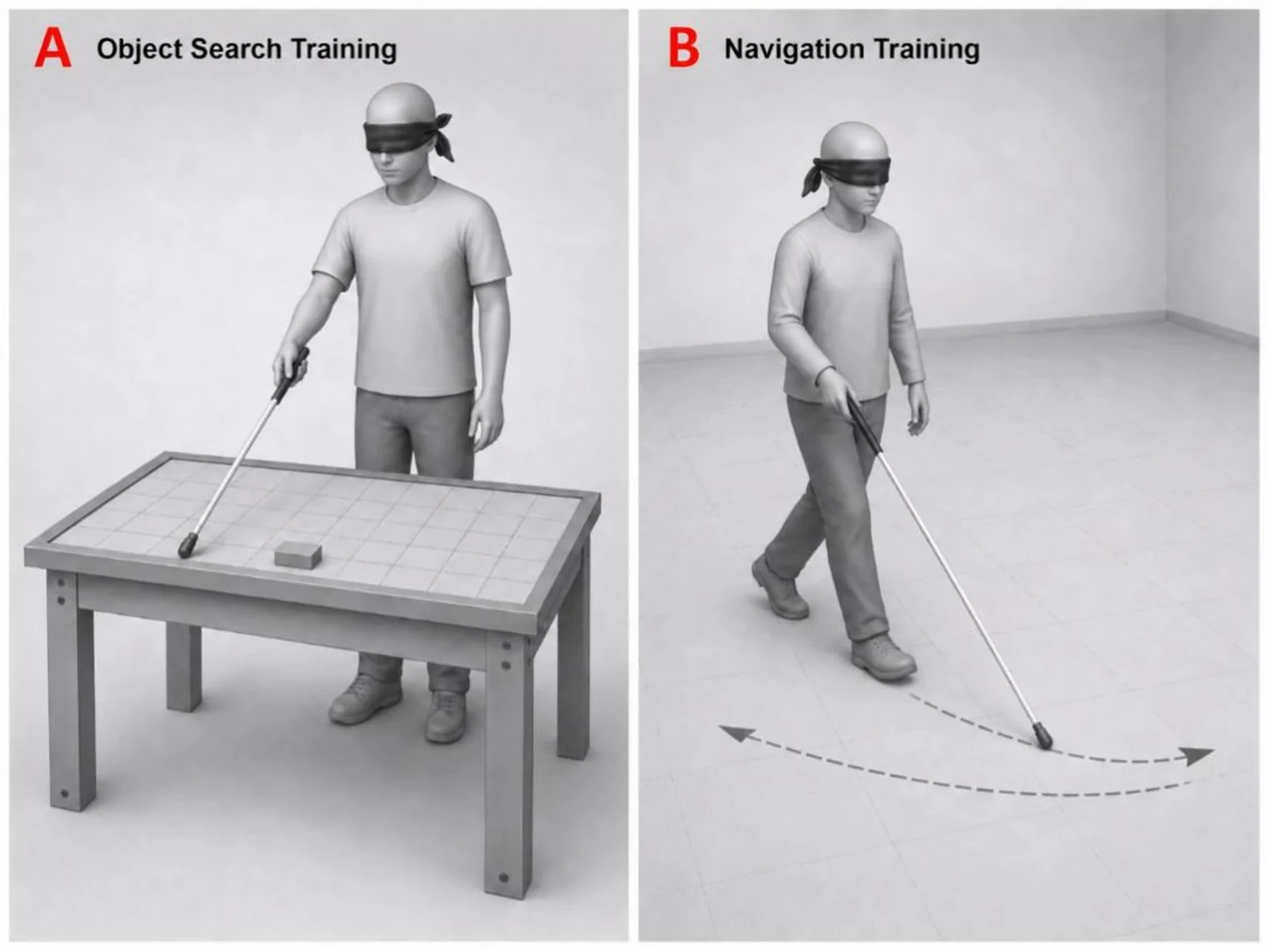
Schematic illustration of the two blindfolded cane-use training conditions. **A** Object Search Training: participants stood 10 cm away from the table edge and used the same 120-cm mobility cane with the dominant right hand to locate a wooden target block on a tabletop grid. **B** Navigation Training: participants used the same 120-cm mobility cane during blindfolded locomotion in a cleared 6 × 5 m room. The same cane was used in both conditions so that differences between conditions reflected functional task demands rather than differences in tool length, weight, or mechanical properties. Note: Figure was generated using ChatGPT v5.5.

The active training blocks were governed by strict, age-dependent time limits to protect participant safety and prevent motor fatigue or secondary complications (e.g., sickness, dizziness, or falls). For the younger adult cohort, the duration for each respective training block was capped at a maximum of 20 min. For the older adult cohort, the training block duration was restricted to a maximum of 10 min. The experimenter closely monitored participants at all times during active locomotion to ensure absolute safety within the visually deprived testing environment (Fig. 2).

### Measurements of tactile distance estimation and reaching distance estimation

#### Tactile distance judgment task measured changes in body schema

TDJ was used to investigate changes in the tactile distance estimation as index of body schema. TDJ task was applied as pre-test and post-test after each training block. TDJ was adapted from recent studies (Jahanian Najafabadi et al. 2023a, b; Jahanian Najafabadi et al. 2026a, b; Jahanian Najafabadi and Godde 2025). Four target body parts (the dorsal surface of the right hand, right forearm, right shin, and right foot) were measured as those body parts were assessed by Sun and Tang (Sun and Tang 2019). During this task, participants comfortably sat on a chair and placed their right hand on the table, while also placing their right leg on the other chair to administer TDJ stimuli.

**Fig. 3:**
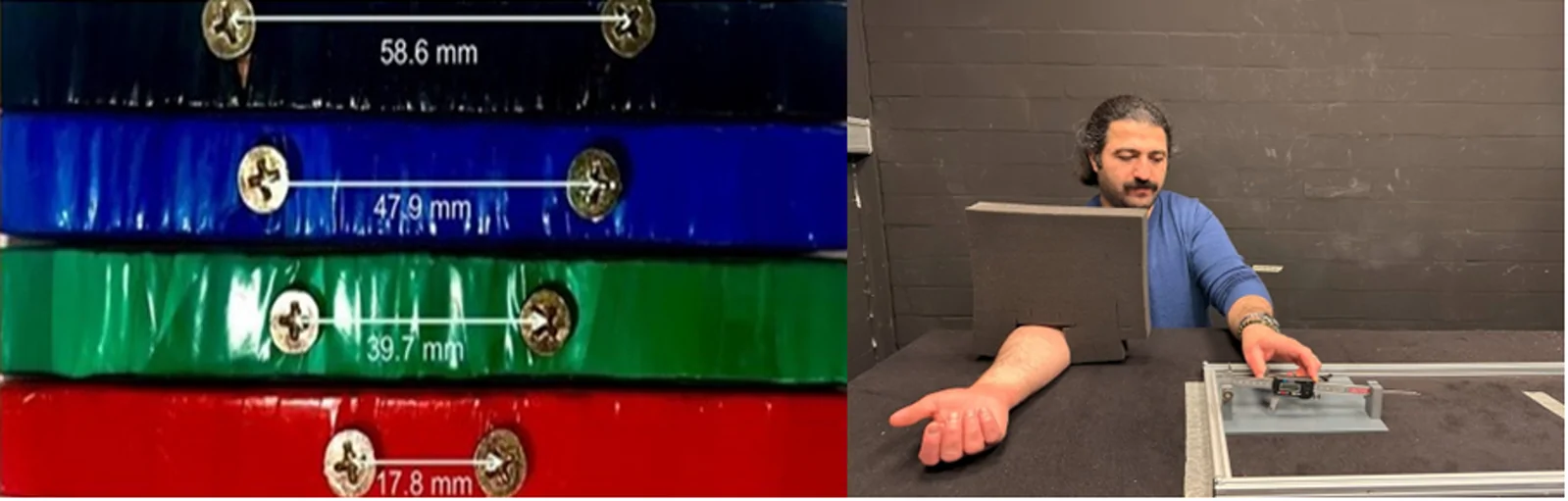
Schematic layout of the Tactile Distance Judgment (TDJ) task configuration used to assess body schema plasticity through measurement of perceived forearm tactile distance. Stimuli were oriented exclusively along the proximodistal (longitudinal) axis of each limb. To maintain complete methodological consistency with the active training configuration, all tactile blocks were executed on the right-sided extremities of blindfolded participants while they rested in a stable, standardized posture

Wooden blocks were prepared with 4 sample pairs of screws, each with different distances between them. The screws had round tips with 9 mm diameter. For TDJ testing parallel to the arm axis (“proximodistal” alignment), 4 sample pairs with distances of 17.8 (Short), 39.7 (Medium), 47.9 (Large), and 58.6 mm (Extra-Large) were administered randomly (cf., Fig. 3). Each trial lasted approximately 1 s. Each sample was presented 3 times, resulting in 12 trials in total (3 trials each per 4 distances).

The participants were additionally asked to report absolute distance estimates, similar to our previous studies (Jahanian Najafabadi et al. 2023a, b: *Experimental brain Research*). Two steel legs were fixed to a piece of wood, which in its turn was attached to the table close to the participant’s left hand, to form a caliper. The participants were able to adjust the distance between the legs with ease. With their left hand, they would then report the perceived distance by adjusting the distance between the caliper legs. This would be displayed on the caliper monitor through a mm scale and would be noted down by the experimenter accordingly (cf., Fig. 3). The stimuli presented to the forearms of the participants would be invisible to the latter due to a restrictive object being placed between their eyes and the right forearm or other target body parts throughout the duration of the TDJ test. The goal of this measure was to ensure no visual information would be obtained about the real distances, and whether the pairs of stimuli were administered on proximodistal orientation.

#### Reaching distance estimation (RDE) task

The RDE task protocol was adapted from prior paradigms (D’Angelo et al. 2018; Jahanian Najafabadi, Rastegari, Imani, et al., 2026Jahanian Najafabadi et al. 2026a, b; Jahanian Najafabadi and Kayser 2026) to evaluate real-time changes in estimated reachability (Bourgeois et al. 2014; Witt et al. 2005). Crucially, while the active training blocks were completed under total visual deprivation, the pre- and post-test RDE measurements were performed with open vision. A small target ball (40 mm in diameter), controlled by the experimenter, moved dynamically along the participant’s midsagittal plane within full view. Because explicit reachability estimates are intrinsically scaled relative to the actor’s physical dimensions (Jahanian Najafabadi, Rastegari, Imani, et al., 2026; Jahanian Najafabadi and Godde 2025), an individual’s true biological arm length acts as a structural confound. Therefore, to isolate the cognitive and plastic components of spatial remapping, each participant’s measured arm length was subtracted from their raw spatial estimations, neutralizing physical limb length as a confounding variable following Bourgeois et al. (Bourgeois et al. 2014).

The target ball’s starting position was set at 33 cm from the participant’s sternum during withdrawing trials, and at 133 cm during approaching trials. These opposing movement directions allowed us to balance out how initial object positioning and directional movement influence reachability judgments (Carello et al. 1989; Rochat and Wraga 1997). During the task, participants stood with their hands resting loosely behind their bodies. To ensure maximum psychophysical precision, participants watched the target move and were permitted to fine-tune the final boundary estimate by verbally instructing the experimenter to nudge the actuator slightly forward or backward.

The task comprised 6 trials in total, counterbalancing approaching (3 trials) and withdrawing (3 trials) sequences. Participants were given explicit, standardized instructions: “This target will now move relative to your position. Please say stop at the exact moment you perceive that you could just barely touch it with your right arm fully outstretched.” To prevent participants from gaining absolute spatial anchor hints or tracking the physical baseline adjustments between tasks, they were instructed to briefly close their eyes between sequential trials while the experimenter silently reset the linear actuator’s starting location.

The physical distance from the tip of the linear actuator to the participant was recorded at the verbal boundary decision. This raw measurement was converted to absolute distance from the sternum, and the participant’s physical arm length was subtracted from the mean score of the 6 trials, yielding a final overall distance estimation error (in cm). Positive error scores indicate an overestimation of reachability (reflecting a perceived expansion of action space beyond the physical hand), whereas negative error scores indicate an underestimation of reachability. Crucially, because participants performed the visual RDE tasks using only their bare arm without holding the tool, post-training shifts in the RDE error index specifically reflect the enduring, offline calibration of reachable action space boundaries triggered by the preceding blindfolded tool use block. The final RDE error scores were used for further statistical analysis.

#### Explicit measures of ownership and agency

To quantify subjective tool embodiment, we adapted the explicit ownership and agency questionnaire design originally introduced by prior researchers (Kalckert and Ehrsson 2012, 2014; Zhang and Hommel 2016), which has been subsequently implemented in virtual and physical tool use paradigms (e.g., Jahanian Najafabadi et al. 2023a, 2025, 2026a, b), see Table 1. To ensure participants did not misinterpret the semantic structure of the assertions—thereby maximizing data reliability—each statement was evaluated using a Visual Analog Scale, bounded by the anchors “Strongly Disagree” and “Strongly Agree”. Participants were instructed to make a single vertical mark along a continuous 100-mm line to denote their level of agreement. These spatial marks were subsequently digitized and transformed onto a standard metric scale ranging from − 50 to + 50 for statistical analyses which has been already implemented in our recent research (Jahanian Najafabadi et al. 2026a, b).

Methodologically, utilizing a continuous analog scale instead of categorical ordinal measurements yields highly individualized psychophysical profiles. By affording participants absolute flexibility along a continuous spectrum, a Visual Analog Scale significantly minimizes the central tendency and extreme-choice biases frequently observed in traditional Likert scales (Grünbaum and Christensen 2020; Kuhlmann et al. 2017; Wenke et al. 2010). This finer measurement granularity is vital for capturing subtle, incremental shifts in explicit body metrics, which are typical of short-term non-biomorphic tool acquisition protocols.

To facilitate cross-study compatibility with foundational embodiment literature, we established a mathematical threshold benchmark for the emergence of a positive embodiment experience. Prior landmarks (Kalckert and Ehrsson 2012, 2012), traditionally employ an ordinal 7-point Likert scale ranging from − 3 to + 3, adopting a conservative score of ≥ + 1 to verify that an individual experienced a meaningful subjective sense of ownership or agency. To map this benchmark directly onto our continuous metrics, we calculated the exact proportional equivalent of this threshold within our − 50 to + 50 parameter workspace. A score of + 1 on a 7-point scale (where 0 represents absolute neutrality) corresponds to the upper 16.7% zone of positive agreement. Extrapolating this identical mathematical ratio onto our 50-unit positive vector space [50 × 1/3 = 16.67) yielded a precise threshold value of + 17. Consequently, a cumulative score of + 17 or above was utilized as our explicit empirical benchmark indicating that a participant experienced a robust, cross-comparable emergence of either explicit tool ownership or behavioral agency.

**Table 1:** Statements used in the ownership and agency questionnaire (used in e.g., Jahanian Najafabadi et al. 2023a, b, 2025)

Variable	Statement
Ownership	Q1: I felt as if the cane was an extension of my own hand
	Q2: I felt as if the cane was part of my body
	Q3: I felt as if the cane was my hand
Ownership-related	Q4: It seems as if I had more than one right hand
	Q5: It felt as if my right hand no longer mattered, as if I only needed to sense the cane
	Q6: I felt as if my real hand developed an enhanced sense of virtual touch
Agency	Q7: I felt as if I could cause movements of the cane
	Q8: I felt as if I could control movements of the cane
	Q9: The cane was obeying my will and I could make it move just like I wanted it to
Agency-related	Q10: I felt as if the cane was controlling my movements
	Q11: It seemed as if the cane had a will of its own
	Q12: I felt as if the cane was controlling me

### Statistical analysis

Inferential statistical analyses were performed using Python v3.13. Data handling and participant-level aggregation were conducted with pandas and numpy, and regression models were fitted using ordinary least squares in statsmodels. The alpha level was set at *p* < .05, two-tailed. Values between 0.05 and 0.10 were described, where relevant, as non-significant trends and were not interpreted as statistically significant.

Behavioral pre-post changes were analyzed using mixed-design ANOVA models with Age Group as a between-subjects factor and Session as a within-subjects factor. These analyses were conducted to evaluate whether perceived forearm tactile distance and perceived distance estimation differed between younger and older adults. Because training duration was not fully equated across age groups for safety reasons, age group comparisons were interpreted cautiously. Younger adults completed longer training blocks than older adults, whereas older adult training duration was reduced to minimize fatigue, discomfort, dizziness, and fall risk during blindfolded navigation.

To examine whether subjective ownership and agency were associated with behavioral recalibration measures, hierarchical linear regression models were fitted separately for each age group, condition, and outcome. This statistical procedure followed similar data analyses approach conducted in prior research (Jahanian Najafabadi et al. 2023a, b, 2026a, b). Ownership, ownership-related, agency, and agency-related ratings were analyzed as dependent variables. In Model 1, TDJ residual estimation error was entered as the sole predictor, indexing forearm tactile distance estimation. In Model 2, RDE error and the TDJ Error × RDE Error interaction were added, indexing perceived reaching distance estimation and its interaction with perceived forearm tactile distance. The comparison between Model 1 and Model 2 tested whether RDE error and the interaction term explained additional variance beyond TDJ error alone. The *p* value reported for the Model 2 comparison refers to this hierarchical model-comparison test, whereas coefficient-level *p* values refer to individual predictors within the full model.

For the joint age group analyses, participant level regression models were fitted separately for each condition and outcome. These models included Age Group, standardized TDJ error, standardized RDE error, the two-way interactions Age Group × TDJ Error, Age Group × RDE Error, and TDJ Error × RDE Error, and the three-way interaction Age Group × TDJ Error × RDE Error. These models tested whether the association between behavioral recalibration measures and subjective ownership or agency differed between younger and older adults.

Effect sizes were reported for both significant and non-significant effects. For ANOVA-based analyses, partial eta squared ηp² was reported. For pairwise comparisons, Cohen’s *d* was reported where applicable. For regression analyses, model fit was reported using *R*² and adjusted *R*², and model-level effect size was quantified using Cohen’s *f*², calculated as *R*²/(1 − *R*²). For hierarchical regression models, the incremental contribution of added predictors was reported using Δ*R*² and Δ*f*², calculated as (*R*²Model 2 − *R*²Model 1)/(1 − *R*²Model 2). Individual regression predictors were reported with coefficients, standard errors, 95% confidence intervals, and *p* values. Cohen’s *f*² values of 0.02, 0.15, and 0.35 were interpreted as small, medium, and large effects, respectively (Lachenbruch and Cohen 1989).

Because no formal a priori power analysis was conducted, a sensitivity power analysis was performed to estimate the minimum detectable effects for the available sample. For the between-group comparison between younger and older adults, the available sample provided approximately 80% power to detect large effects of about Cohen’s *d* = 0.91 at α = 0.05, two-tailed. Within the older adult group, *n* = 16 provided approximately 80% power to detect within-participant effects of about Cohen’s *d* = 0.75. For the participant-level joint regression models, the study was mainly sensitive to large overall model effects, approximately Cohen’s *f*^2^ = 0.40–0.42, and to medium-to-large individual predictor or interaction effects, approximately partial *f*² = 0.19–0.20. Therefore, non-significant small effects, particularly higher order age interactions, were interpreted cautiously rather than as evidence for the absence of an effect.

## Results

### Tool-body ownership and sense of agency

#### Younger adults

In Condition A, mean ratings of − 8.46 (SE = 2.94) and − 1.76 (SE = 2.07) were obtained for ownership and ownership-related items, respectively, whereas mean ratings of 32.60 (SE = 1.85) and 28.48 (SE = 2.05) were obtained for agency and agency-related items. Thus, only agency and agency-related ratings, but not ownership or ownership-related ratings, were above the + 17 positive-endorsement benchmark derived from Kalckert and Ehrsson (Kalckert and Ehrsson 2012, 2014).

In Condition B, mean ratings of 7.50 (SE = 2.82) and − 2.74 (SE = 2.15) were obtained for ownership and ownership-related items, respectively, whereas mean ratings of 34.83 (SE = 1.20) and 20.15 (SE = 2.29) were obtained for agency and agency-related items. Thus, as in Condition A, only agency and agency-related ratings exceeded the + 17 benchmark, whereas ownership and ownership-related ratings did not.

Hierarchical linear regressions were conducted to examine whether subjective ownership and agency ratings were associated with perceived forearm tactile distance and perceived reaching distance estimation. For each outcome, Model 1 included TDJ residual estimation error as the sole predictor. Model 2 added RDE error and the TDJ Error × RDE Error interaction. The Model 2 comparison tested whether adding RDE error and the interaction term improved prediction beyond TDJ error alone.

### Condition A

For ownership, Model 1 was not statistically significant, *R*² = 0.007, adjusted *R*² = −0.002, *F*(1, 106) = 0.76, *p* = .386, Cohen’s *f*² = 0.007. TDJ residual estimation error did not significantly predict ownership, *p* = .386. Adding RDE error and the TDJ Error × RDE Error interaction significantly improved model fit, Δ*R*² = 0.074, Δ*f*² = 0.081, *F*(2, 104) = 4.20, *p* = .018. The full Model 2 was statistically significant, *R*² = 0.081, adjusted *R*² = 0.055, *F*(3, 104) = 3.07, *p* = .031, Cohen’s *f*² = 0.089. In Model 2, RDE error significantly predicted ownership, *B* = − 0.77, SE = 0.33, 95% CI [− 1.42, − 0.13], *p* = .020. TDJ residual estimation error, *p* = .284, and the TDJ Error × RDE Error interaction, *p* = .861, were not significant predictors.

For ownership-related ratings, Model 1 was not statistically significant, *R*² = 0.018, adjusted *R*² = 0.008, *F*(1, 106) = 1.89, *p* = .172, Cohen’s *f*² = 0.018. TDJ residual estimation error did not significantly predict ownership-related ratings, *p* = .172. Adding RDE error and the TDJ Error × RDE Error interaction did not significantly improve model fit, Δ*R*² = 0.036, Δ*f*² = 0.038, *F*(2, 104) = 1.97, *p* = .144. The full Model 2 was not statistically significant, *R*² = 0.053, adjusted *R*² = 0.026, *F*(3, 104) = 1.96, *p* = .125, Cohen’s *f*² = 0.056. None of the individual predictors reached statistical significance. The TDJ Error × RDE Error interaction showed a non-significant trend, *B* = − 0.027, SE = 0.015, 95% CI [− 0.057, 0.003], *p* = .079.

For agency, Model 1 was not statistically significant, *R*² = 0.015, adjusted *R*² = 0.005, *F*(1, 106) = 1.58, *p* = .211, Cohen’s *f*² = 0.015. TDJ residual estimation error did not significantly predict agency, *p* = .211. Adding RDE error and the TDJ Error × RDE Error interaction did not improve model fit, Δ*R*² = 0.004, Δ*f*² = 0.004, *F* (2, 104) = 0.23, *p* = .793. The full Model 2 was not statistically significant, *R*² = 0.019, adjusted *R*² = −0.009, *F*(3, 104) = 0.67, *p* = .570, Cohen’s *f*² = 0.019. No individual predictor was statistically significant.

For agency-related ratings, Model 1 was not statistically significant, *R*² = 0.003, adjusted *R*² = −0.006, *F*(1, 106) = 0.32, *p* = .572, Cohen’s *f*² = 0.003. TDJ residual estimation error did not significantly predict agency-related ratings, *p* = .572. Adding RDE error and the TDJ Error × RDE Error interaction did not significantly improve model fit, Δ*R*² = 0.034, Δ*f*² = 0.035, *F*(2, 104) = 1.82, *p* = .168. The full Model 2 was not statistically significant, *R*² = 0.037, adjusted *R*² = 0.009, *F*(3, 104) = 1.32, *p* = .272, Cohen’s *f*² = 0.038. TDJ residual estimation error, *B* = − 0.89, SE = 0.46, 95% CI [− 1.80, 0.02], *p* = .056, and the TDJ Error × RDE Error interaction, *B* = − 0.029, SE = 0.015, 95% CI [− 0.058, 0.001], *p* = .060, showed non-significant trends. RDE error was not significant, *p* = .208. Overall, in Condition A, perceived reaching distance significantly predicted ownership, whereas agency and agency-related ratings were not reliably explained by TDJ or RDE measures.

### Condition B

For ownership, Model 1 was not statistically significant, *R*² = 0.020, adjusted *R*² = 0.010, *F*(1, 102) = 2.04, *p* = .157, Cohen’s *f*² = 0.020. TDJ residual estimation error did not significantly predict ownership, *p* = .157. Adding RDE error and the TDJ Error × RDE Error interaction did not significantly improve model fit, Δ*R*² = 0.017, Δ*f*² = 0.018, *F* (2, 100) = 0.88, *p* = .420. The full Model 2 was not statistically significant, *R*² = 0.036, adjusted *R*² = 0.008, *F*(3, 100) = 1.26, *p* = .292, Cohen’s *f*² = 0.038. No individual predictor was statistically significant.

For ownership-related ratings, Model 1 was not statistically significant, *R*² = 0.024, adjusted *R*² = 0.014, *F*(1, 102) = 2.48, *p* = .118, Cohen’s *f*² = 0.024. TDJ residual estimation error did not significantly predict ownership-related ratings, *p* = .118. Adding RDE error and the TDJ Error × RDE Error interaction significantly improved model fit, Δ*R*² = 0.064, Δ*f*² = 0.070, *F*(2, 100) = 3.49, *p* = .034. The full Model 2 was statistically significant, *R*² = 0.088, adjusted *R*² = 0.060, *F*(3, 100) = 3.20, *p* = .027, Cohen’s *f*² = 0.096. In Model 2, TDJ residual estimation error significantly predicted ownership-related ratings, *B* = − 1.15, SE = 0.55, 95% CI [− 2.25, − 0.05], *p* = .040. RDE error also significantly predicted ownership-related ratings, *B* = 0.46, SE = 0.17, 95% CI [0.11, 0.80], *p* = .010. The TDJ Error × RDE Error interaction was not significant, *p* = .149.

For agency, Model 1 was not statistically significant, *R*² = 0.006, adjusted *R*² = −0.003, *F*(1, 102) = 0.65, *p* = .423, Cohen’s *f*² = 0.006. TDJ residual estimation error did not significantly predict agency, *p* = .423. Adding RDE error and the TDJ Error × RDE Error interaction significantly improved model fit, Δ*R*² = 0.075, Δ*f*² = 0.081, *F* (2, 100) = 4.07, *p* = .020. The full Model 2 was statistically significant, *R*² = 0.081, adjusted *R*² = 0.054, *F*(3, 100) = 2.94, *p* = .037, Cohen’s *f*² = 0.088. In Model 2, TDJ residual estimation error significantly predicted agency, *B* = − 0.67, SE = 0.31, 95% CI [− 1.29, − 0.06], *p* = .032. RDE error also significantly predicted agency, *B* = 0.27, SE = 0.10, 95% CI [0.08, 0.46], *p* = .006. The TDJ Error × RDE Error interaction showed a non-significant trend, *B* = − 0.018, SE = 0.009, 95% CI [− 0.035, 0.0004], *p* = .056.

For agency-related ratings, Model 1 was not statistically significant, *R*² = 0.004, adjusted *R*² = −0.006, *F*(1, 102) = 0.44, *p* = .510, Cohen’s *f*² = 0.004. TDJ residual estimation error did not significantly predict agency-related ratings, *p* = .510. Adding RDE error and the TDJ Error × RDE Error interaction did not significantly improve model fit, Δ*R*² = 0.032, Δ*f*² = 0.033, *F* (2, 100) = 1.64, *p* = .200. The full Model 2 was not statistically significant, *R*² = 0.036, adjusted *R*² = 0.007, *F*(3, 100) = 1.24, *p* = .300, Cohen’s *f*² = 0.037. No individual predictor was statistically significant.

Overall, in Condition B, ownership itself was not reliably predicted by TDJ or RDE measures. However, ownership-related ratings and agency were significantly predicted by the full Model 2, with both TDJ residual estimation error and RDE error contributing as significant predictors. Thus, during navigation, subjective agency and ownership-related ratings were more closely associated with behavioral recalibration measures than during object search.

### Older adults

In **Condition A**, mean ratings of − 0.29 (SE = 3.00) and 0.56 (SE = 2.98) were obtained for ownership and ownership-related items, respectively, whereas mean ratings of 24.12 (SE = 2.77) and 23.75 (SE = 3.20) were obtained for agency and agency-related items. Thus, only agency and agency-related ratings, but not ownership or ownership-related ratings, exceeded the + 17 positive-endorsement benchmark derived from Kalckert and Ehrsson (2012, 2014).

In **Condition B**, mean ratings of 16.63 (SE = 2.08) and − 2.08 (SE = 2.59) were obtained for ownership and ownership-related items, respectively, whereas mean ratings of 18.98 (SE = 3.24) and 25.21 (SE = 2.95) were obtained for agency and agency-related items. Thus, agency and agency-related ratings exceeded the + 17 benchmark, whereas ownership was close to but did not clearly exceed the benchmark, and ownership-related ratings remained below it.

Hierarchical linear regressions were conducted to examine whether subjective ownership and agency ratings were associated with perceived forearm tactile distance and perceived reaching distance. For each outcome, Model 1 included TDJ residual estimation error as the sole predictor. Model 2 added RDE error and the TDJ Error × RDE Error interaction.

In **Condition A**, For ownership, Model 1 was statistically significant, *R*² = 0.134, adjusted *R*² = 0.120, *F*(1, 62) = 9.57, *p* = .003, Cohen’s *f*² = 0.154. TDJ residual estimation error significantly predicted ownership in Model 1. Adding RDE error and the TDJ Error × RDE Error interaction did not significantly improve model fit, Δ*R*² = 0.011, Δ*f*² = 0.013, *F*(2, 60) = 0.39, *p* = .680. The full Model 2 was statistically significant, *R*² = 0.145, adjusted *R*² = 0.102, *F*(3, 60) = 3.39, *p* = .024, Cohen’s *f*² = 0.169. However, none of the individual predictors in Model 2 reached statistical significance.

For ownership-related ratings, Model 1 was not statistically significant, *R*² = 0.001, adjusted *R*² = −0.015, *F*(1, 62) = 0.05, *p* = .832, Cohen’s *f*² = 0.001. TDJ residual estimation error did not significantly predict ownership-related ratings. Adding RDE error and the TDJ Error × RDE Error interaction did not improve model fit, Δ*R*² = 0.013, Δ*f*² = 0.013, *F* (2, 60) = 0.40, *p* = .670. The full Model 2 was not statistically significant, *R*² = 0.014, adjusted *R*² = −0.035, *F*(3, 60) = 0.28, *p* = .837, Cohen’s *f*² = 0.014. No individual predictor was significant.

For agency, Model 1 was not statistically significant, *R*² = 0.017, adjusted *R*² = 0.001, *F*(1, 62) = 1.05, *p* = .310, Cohen’s *f*² = 0.017. TDJ residual estimation error did not significantly predict agency. Adding RDE error and the TDJ Error × RDE Error interaction significantly improved model fit, Δ*R*² = 0.298, Δ*f*² = 0.436, *F*(2, 60) = 13.07, *p* < .001. The full Model 2 was statistically significant, *R*² = 0.315, adjusted *R*² = 0.281, *F*(3, 60) = 9.20, *p* < .001, Cohen’s *f*² = 0.460. In Model 2, RDE error significantly predicted agency, *B* = 1.99, SE = 0.40, 95% CI [1.19, 2.79], *p* < .001. TDJ residual estimation error, *p* = .423, and the TDJ Error × RDE Error interaction, *p* = .491, were not significant predictors.

For agency-related ratings, Model 1 was not statistically significant, *R*² = 0.021, adjusted *R*² = 0.005, *F*(1, 62) = 1.34, *p* = .252, Cohen’s *f*² = 0.022. TDJ residual estimation error did not significantly predict agency-related ratings. Adding RDE error and the TDJ Error × RDE Error interaction significantly improved model fit, Δ*R*² = 0.219, Δ*f*² = 0.288, *F* (2, 60) = 8.65, *p* < .001. The full Model 2 was statistically significant, *R*² = 0.240, adjusted *R*² = 0.202, *F*(3, 60) = 6.32, *p* < .001, Cohen’s *f*² = 0.316. In Model 2, RDE error significantly predicted agency-related ratings, *B* = 2.02, SE = 0.49, 95% CI [1.05, 2.99], *p* < .001. TDJ residual estimation error, *p* = .724, and the TDJ Error × RDE Error interaction, *p* = .588, were not significant predictors. Overall, in Condition A, ownership was predicted by TDJ residual estimation error in Model 1, but this effect was not retained as a significant individual predictor once RDE error and the interaction term were added. Agency and agency-related ratings were more clearly associated with perceived reaching distance.

In **Condition B**, for ownership, Model 1 was not statistically significant, *R*² = 0.008, adjusted *R*² = −0.008, *F*(1, 62) = 0.50, *p* = .484, Cohen’s *f*² = 0.008. TDJ residual estimation error did not significantly predict ownership. Adding RDE error and the TDJ Error × RDE Error interaction did not improve model fit, Δ*R*² = 0.001, Δ*f*² = 0.001, *F* (2, 60) = 0.02, *p* = .984. The full Model 2 was not statistically significant, *R*² = 0.008, adjusted *R*² = −0.041, *F*(3, 60) = 0.17, *p* = .916, Cohen’s *f*² = 0.009. No individual predictor was significant.

For ownership-related ratings, Model 1 was not statistically significant, *R*² = 0.007, adjusted *R*² = −0.009, *F*(1, 62) = 0.43, *p* = .516, Cohen’s *f*² = 0.007. TDJ residual estimation error did not significantly predict ownership-related ratings. Adding RDE error and the TDJ Error × RDE Error interaction did not significantly improve model fit, Δ*R*² = 0.063, Δ*f*² = 0.068, *F* (2, 60) = 2.05, *p* = .138. The full Model 2 was not statistically significant, *R*² = 0.070, adjusted *R*² = 0.024, *F*(3, 60) = 1.51, *p* = .221, Cohen’s *f*² = 0.076. No individual predictor reached statistical significance. TDJ residual estimation error, *B* = 1.57, SE = 0.82, 95% CI [− 0.08, 3.21], *p* = .062, and the TDJ Error × RDE Error interaction, *B* = 0.058, SE = 0.033, 95% CI [− 0.008, 0.125], *p* = .085, showed non-significant trends.

For agency, Model 1 was statistically significant, *R*² = 0.065, adjusted *R*² = 0.050, *F*(1, 62) = 4.31, *p* = .042, Cohen’s *f*² = 0.070. TDJ residual estimation error significantly predicted agency in Model 1. Adding RDE error and the TDJ Error × RDE Error interaction significantly improved model fit, Δ*R*² = 0.297, Δ*f*² = 0.465, *F* (2, 60) = 13.94, *p* < .001. The full Model 2 was statistically significant, *R*² = 0.362, adjusted *R*² = 0.330, *F*(3, 60) = 11.33, *p* < .001, Cohen’s *f*² = 0.566. In Model 2, TDJ residual estimation error significantly predicted agency, *B* = 1.81, SE = 0.85, 95% CI [0.10, 3.51], *p* = .038. RDE error also significantly predicted agency, *B* = 1.60, SE = 0.37, 95% CI [0.85, 2.34], *p* < .001. The TDJ Error × RDE Error interaction showed a non-significant trend, *B* = 0.060, SE = 0.034, 95% CI [− 0.009, 0.129], *p* = .085.

For agency-related ratings, Model 1 was not statistically significant, *R*² = 0.005, adjusted *R*² = −0.011, *F*(1, 62) = 0.30, *p* = .584, Cohen’s *f*² = 0.005. TDJ residual estimation error did not significantly predict agency-related ratings. Adding RDE error and the TDJ Error × RDE Error interaction significantly improved model fit, Δ*R*² = 0.304, Δ*f*² = 0.441, *F* (2, 60) = 13.22, *p* < .001. The full Model 2 was statistically significant, *R*² = 0.309, adjusted *R*² = 0.275, *F*(3, 60) = 8.95, *p* < .001, Cohen’s *f*² = 0.448. In Model 2, RDE error significantly predicted agency-related ratings, *B* = 1.68, SE = 0.35, 95% CI [0.97, 2.38], *p* < .001. TDJ residual estimation error, *p* = .629, and the TDJ Error × RDE Error interaction, *p* = .587, were not significant predictors. Overall, in Condition B, ownership and ownership-related ratings were not reliably predicted by TDJ or RDE measures. In contrast, agency and agency-related ratings were significantly associated with behavioral recalibration measures, especially perceived reaching distance. This suggests that, in older adults, subjective control over the cane during navigation was more strongly related to reachability updating than explicit ownership over the tool.

### Joint analyses

To examine whether the associations between behavioral recalibration measures and subjective tool experience differed by age group, we conducted joint regression analyses including both younger and older adults. These analyses focused on the primary ownership and agency outcomes. Data analyses were performed at the participant level. For each participant and condition, TDJ residual estimation error was averaged across tactile-distance levels, whereas RDE error and ownership/agency ratings were retained as participant-level measures. Predictors were z-standardized within each condition, and Age Group. Separate models were fitted for ownership and agency in each condition using the full interaction model: Outcome ~ TDJ Error × RDE Error × Age Group. Model-level effect size was quantified using Cohen’s *f*², and term-level effects were reported using partial η².

In **Condition A**, object search training, the ownership model was not statistically significant, *F*(7, 35) = 0.93, *p* = .494, *R*² = 0.157, adjusted *R*² = −0.011, Cohen’s *f*² = 0.186. None of the predictors or interaction terms reached statistical significance, including Age Group, TDJ Error, RDE Error, or the TDJ Error × RDE Error × Age Group interaction. Thus, ownership ratings during object search were not reliably explained by perceived forearm tactile distance, perceived reaching distance estimation, or their age-dependent interactions.

**Fig. 4:**
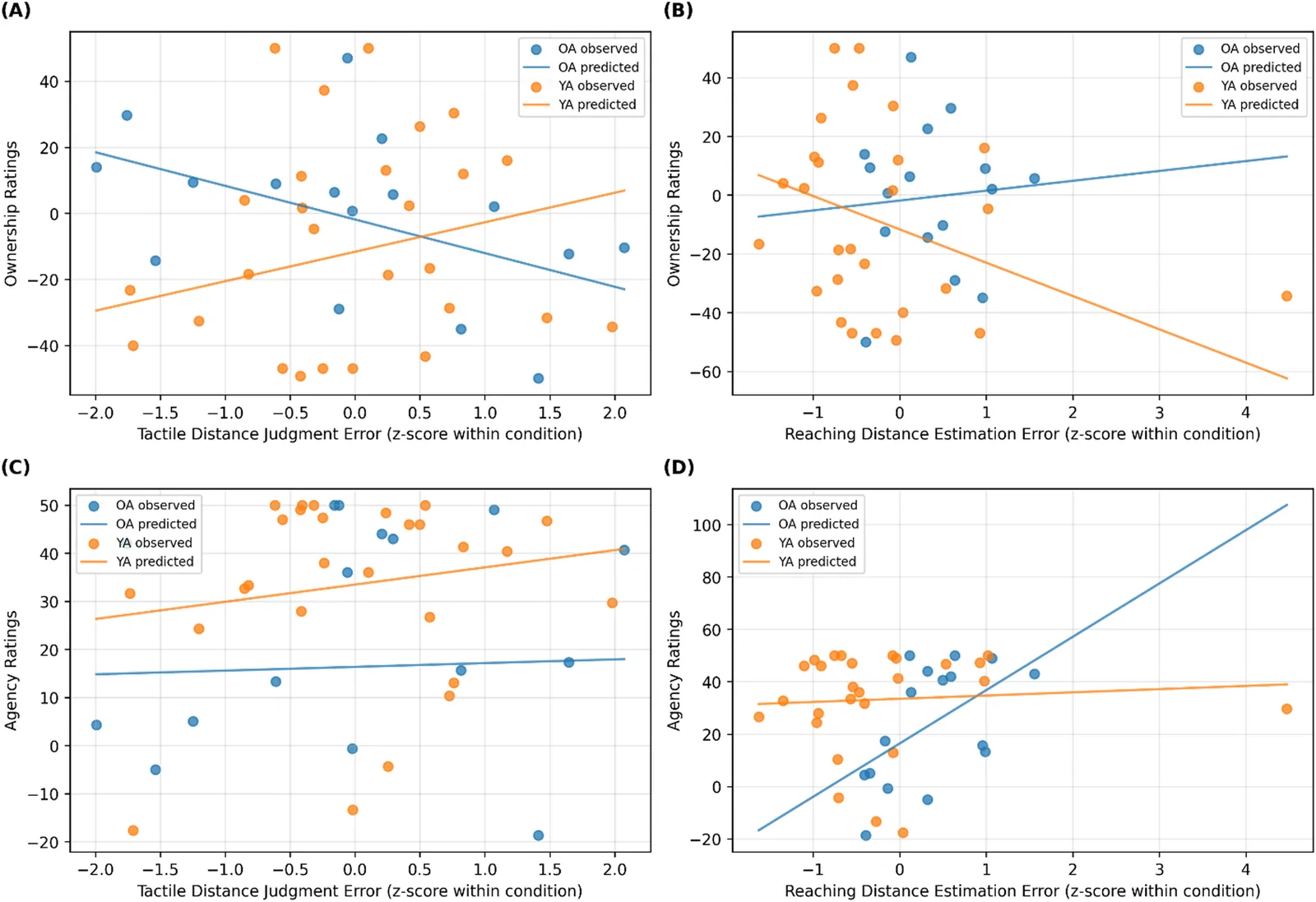
Separate predictor plots for the joint regression model in Condition A. Panel (**A**) shows the association between TDJ Error and ownership ratings, with RDE Error held at its mean value. Panel (**B**) shows the association between Reaching Distance Estimation (RDE) Error and ownership ratings, with TDJ Error held at its mean value. Panel (**C**) shows the association between TDJ Error and agency ratings, with RDE Error held at its mean value. Panel (**D**) shows the association between RDE Error and agency ratings, with TDJ Error held at its mean value. Points represent observed participant-level data for younger adults (YA) and older adults (OA), and lines represent model-predicted values from the joint regression model including TDJ Error, RDE Error, Age Group, and their interactions. TDJ Error and RDE Error were z-standardized within condition

In **Condition A**, agency model also did not reach statistical significance, *F*(7, 35) = 1.19, *p* = .333, *R*² = 0.192, adjusted *R*² = 0.031, Cohen’s *f*² = 0.238. Within this non-significant overall model, RDE Error showed a significant term-level association with agency, *F*(1, 35) = 4.81, *p* = .035, partial η² = 0.121, and Age Group also showed a significant term-level effect, *F*(1, 35) = 4.79, *p* = .035, partial η² = 0.120. However, the RDE Error × Age Group interaction did not reach statistical significance, *F*(1, 35) = 2.97, *p* = .094, partial η² = 0.078, and the three-way TDJ Error × RDE Error × Age Group interaction was not significant, *F*(1, 35) = 0.36, *p* = .553, partial η² = 0.010. Therefore, Condition A provided only limited evidence that perceived reaching distance was associated with agency, and no reliable evidence that this association differed by age group. As shown in Fig. 4, separate predictor plots were used to visualize the joint regression model for Condition A. Neither ownership nor agency was reliably explained by the full joint model in this condition, although agency showed a limited term-level association with RDE Error and Age Group.

In **Condition B**, navigation training, the ownership model was not statistically significant, *F*(7, 34) = 0.37, *p* = .912, *R*² = 0.071, adjusted *R*² = −0.120, Cohen’s *f*² = 0.077. No main effects or interaction terms reached significance. Thus, ownership ratings during navigation were not reliably predicted by TDJ Error, RDE Error, Age Group, or age-related moderation of these predictors.

**Fig. 5:**
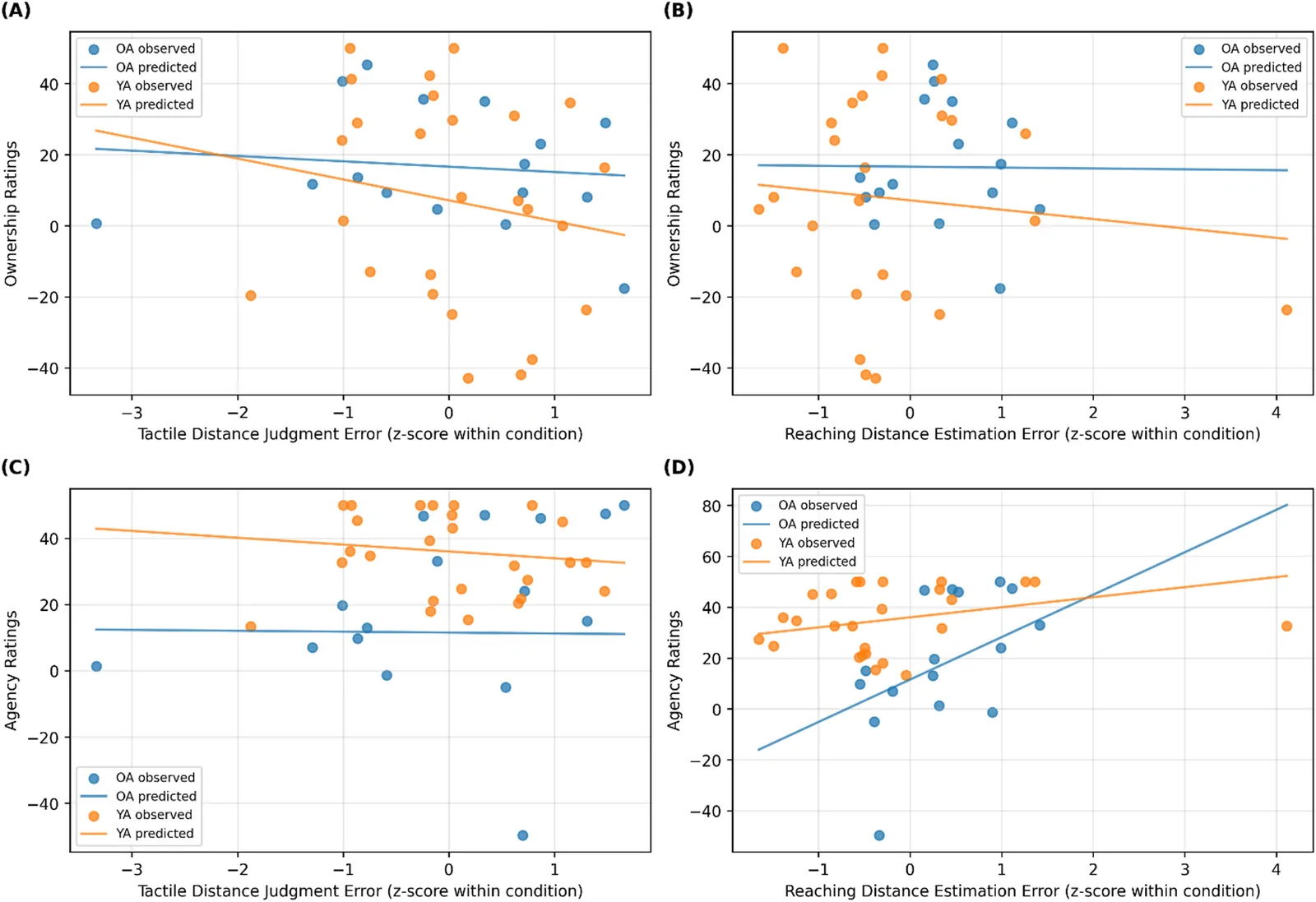
Separate predictor plots for the joint regression model in Condition B. Panel (**A**) shows the association between TDJ Error and ownership ratings, with RDE Error held at its mean value. Panel (**B**) shows the association between Reaching Distance Estimation (RDE) Error and ownership ratings, with TDJ Error held at its mean value. Panel (**C**) shows the association between TDJ Error and agency ratings, with RDE Error held at its mean value. Panel (**D**) shows the association between RDE Error and agency ratings, with TDJ Error held at its mean value. Points represent observed participant-level data for younger adults (YA) and older adults (OA), and lines represent model-predicted values from the joint regression model including TDJ Error, RDE Error, Age Group, and their interactions. TDJ Error and RDE Error were z-standardized within condition

In contrast, the Condition B agency model was statistically significant, *F*(7, 34) = 3.79, *p* = .004, *R*² = 0.438, adjusted *R*² = 0.323, Cohen’s *f*² = 0.780. RDE Error significantly predicted agency, *F*(1, 34) = 4.75, *p* = .036, partial η² = 0.123, indicating that variation in perceived reaching distance was associated with agency ratings. Age Group also significantly predicted agency, *F*(1, 34) = 17.06, *p* < .001, partial η² = 0.334, with younger adults reporting higher agency than older adults at the mean predictor values. TDJ Error did not significantly predict agency, *F*(1, 34) = 0.004, *p* = .950, partial η² < 0.001. The TDJ Error × RDE Error interaction did not reach statistical significance, *F*(1, 34) = 3.24, *p* = .081, partial η² = 0.087. Similarly, the TDJ Error × RDE Error × Age Group interaction remained below the significance threshold, *F*(1, 34) = 3.93, *p* = .056, partial η² = 0.104. Thus, agency during navigation was the only outcome reliably explained by the joint model, with the clearest evidence for contributions of perceived reaching distance and Age Group. Overall, the participant-level joint analyses did not support the stronger claim that behavioral recalibration measures robustly predicted explicit ownership. They also did not provide clear evidence that Age Group significantly moderated ownership-related associations. The most reliable joint-analysis finding was observed for agency during navigation: this model explained substantial variance and showed significant effects of RDE Error and Age Group. Evidence for higher-order age-dependent sensorimotor interactions was suggestive but did not reach the conventional significance threshold after correcting the analysis to the participant level. As shown in Fig. 5, separate predictor plots were used to visualize the joint regression model for Condition B. Ownership was not reliably explained by the model, whereas agency was significantly explained by the full joint model, with significant contributions of RDE Error and Age Group.

## Discussion

The present study examined whether younger and older adults experience explicit ownership and agency over a cane during short-term visual deprivation, and whether these experiences differ between two functionally distinct forms of tool use: object search and navigation. A further aim was to determine whether subjective embodiment ratings were associated with behavioral indices of tool-related recalibration, specifically perceived forearm tactile distance recalibration and perceived reaching distance. This distinction is important because explicit ownership and agency do not necessarily reflect the same mechanisms as behavioral changes in perceived forearm tactile distance or reaching distance estimation (Jahanian Najafabadi et al. 2023a; Longo and Serino 2012; Miller et al. 2014; Zanini et al. 2021). Because the present study did not include a sighted control condition, the findings cannot determine whether visual deprivation caused the observed pattern of ownership and agency. Instead, the results show how explicit embodiment and behavioral recalibration are related under blindfolded cane use.

### Ownership and agency during blindfolded cane use

Across both tool use conditions, participants reported stronger agency than ownership over the cane. During object search, both younger and older adults showed weak ownership but positive agency. During navigation, agency again remained the dominant subjective experience, whereas ownership remained low or inconsistent. This pattern suggests that short-term blindfolded cane use was sufficient to support the experience of controlling the tool, but not sufficient to produce a strong conscious feeling that the cane belonged to, or became part of, the body.

This dissociation is theoretically plausible. Agency is closely linked to voluntary action, motor intention, action control, and predicted sensory consequences (Kalckert and Ehrsson 2012; Ma and Hommel 2015; Moore and Fletcher 2012). By contrast, explicit ownership over a non-corporeal object such as a cane is a more demanding subjective experience, because it requires the tool to be experienced as belonging to the body rather than merely being controlled (Kalckert and Ehrsson 2012; Jahanian Najafabadi et al. 2025). Therefore, the present findings should not be interpreted as evidence that visual deprivation selectively reduces ownership more than agency. Such a conclusion would require direct comparison with a sighted tool use condition. Rather, the present results indicate that action-based control over the cane remained subjectively available during blindfolded tool use, whereas explicit ownership over the cane remained weak.

The low ownership ratings are nevertheless informative. Previous work suggests that visual experience and multisensory calibration can shape bodily ownership illusions and tool-related body updating (Nava et al. 2014; Radziun and Ehrsson 2018). However, because the present study did not directly compare sighted and blindfolded tool use, the current results should not be interpreted as showing that vision is necessary for ownership. A more cautious interpretation is that voluntary control of the cane, supported by available nonvisual sensory feedback, was sufficient to support agency over the cane, but did not reliably produce explicit ownership. This interpretation is consistent with the view that agency and ownership are partially separable components of embodiment (Kalckert and Ehrsson 2012; Ma and Hommel 2015).

Importantly, low explicit ownership does not imply the absence of tool-related body or spatial updating. Ownership questionnaires measure conscious endorsement that the tool belongs to, or is part of, the body, whereas TDJ and RDE provide indirect behavioral indices of body schema recalibration and perceived reachability, respectively (Miller et al. 2014; Zanini et al. 2021). Thus, short-term cane use may modulate action-related representations without producing a strong conscious ownership experience. This distinction is consistent with previous work showing that implicit sensorimotor recalibration after tool use can occur without necessarily producing strong explicit embodiment ratings (Cardinali et al. 2009; Jahanian Najafabadi et al. 2023a; Miller et al. 2017b). Thus, the ownership questionnaire should be interpreted as a measure of explicit conscious endorsement of cane ownership, not as a complete measure of implicit tool incorporation.

### Associations between subjective embodiment and behavioral recalibration

The regression analyses suggest that agency was more consistently related to behavioral recalibration measures than ownership. In the separate age group analyses, younger adults showed associations between perceived reaching distance and subjective outcomes across conditions. In Condition A, RDE Error significantly predicted ownership ratings, whereas in Condition B, the full model significantly predicted ownership-related ratings and agency. In older adults, agency ratings were also associated with perceived reaching distance, particularly during navigation. However, the participant-level joint analyses showed that ownership was not reliably predicted by perceived forearm tactile distance, perceived reaching distance, Age Group, or their interactions in either condition. The clearest joint analysis finding was observed for agency during navigation, where the full model was significant and included significant effects of RDE Error and Age Group. This pattern suggests that perceived reaching distance may be more closely related to subjective tool control than perceived forearm tactile distance alone. RDE indexes explicit perceived reachability and action-scaled spatial representation, whereas TDJ indexes local body-metric recalibration (Longo and Haggard 2010; Miller et al. 2014, 2017b; Zanini et al. 2021). The stronger involvement of RDE in agency-related outcomes is therefore consistent with the idea that controlling a cane, especially during navigation, depends on action-scaled estimates of what can be reached, contacted, or explored through the tool. However, these results should not be described as evidence for a general extension of peripersonal space. RDE is best interpreted here as a measure of perceived reachability, not as a direct measure of defensive or multisensory-facilitation peripersonal space (Jahanian Najafabadi, Rastegari, Imani, et al., 2026; Jahanian Najafabadi et al. 2026a, b; Zanini et al. 2021).

The navigation condition may have strengthened the association between behavioral recalibration and agency because it required continuous online control of the cane during blindfolded walking and spatial exploration. Compared with tabletop object search, walking with the cane placed stronger demands on whole-body coordination, action monitoring, proprioceptive feedback, tactile exploration, and continuous spatial updating. Tool use models propose that successful tool use can recalibrate body metrics and action-scaled spatial representations (Cardinali et al. 2012; Martel et al. 2016, 2019). The present findings extend this idea by suggesting that, under blindfolded conditions, such recalibration is more clearly reflected in explicit agency than in ownership.

### Age-related patterns

The age-related findings should be interpreted cautiously. Descriptively, older adults tended to report lower or less consistent embodiment ratings than younger adults, and their behavioral recalibration–agency coupling appeared less stable across models. This pattern is compatible with evidence that aging is associated with changes in proprioceptive reliability, somatosensory processing, visuospatial attention, and action-scaled spatial judgments (Costello and Bloesch 2017; Luyat et al. 2024; Sugovic and Witt 2013). However, the present sample was small and unbalanced, and older adults completed shorter training blocks for safety reasons. Therefore, age group differences cannot be interpreted independently of exposure duration.

The findings are nevertheless consistent with cue-integration accounts of perception and action. These accounts propose that the nervous system weights sensory and motor signals according to their relative reliability (Ernst and Bülthoff 2004). If proprioceptive or somatosensory estimates become less reliable with age, they may contribute less strongly or less consistently to action monitoring and subjective agency. Such an interpretation is compatible with broader accounts of agency in which motor predictions and predicted sensory consequences contribute to the experience of controlling an action (Desantis et al. 2011; Moore et al. 2009; Moore and Fletcher 2012). However, because the study was mainly powered to detect medium-to-large or large effects, small age-related differences and subtle higher-order interactions cannot be ruled out.

This interpretation is consistent with recent lifespan work on intentional or temporal binding, which suggests that the mechanisms supporting agency may change with age. Cavazzana et al. (2017) reported reduced intentional binding in older adults compared with young adults, suggesting that older adults may show weaker implicit agency under conditions requiring attentional control and flexible linking of voluntary action with its sensory consequence (Cavazzana et al. 2017). However, Fujii et al. (2023) reported that older adults showed temporal binding in voluntary action conditions that was comparable to young adults, while also showing strong binding for involuntary actions (Fujii et al. 2023). This pattern suggests that, in older adults, perceived action–effect binding may depend not only on motor intention, but also on causal belief, sensory reliability, and retrospective inference. Therefore, the present finding of preserved explicit agency over the cane should not be interpreted as evidence that motor intention alone is sufficient for agency in older adults. Rather, agency during blindfolded cane use may reflect a combination of voluntary control, nonvisual sensory feedback, action–effect predictability, and causal inference, with the relative weighting of these cues potentially changing with age.

The observed age-related differences in agency ratings, together with the non-significant but suggestive higher-order interaction patterns, may also be relevant to broader theories of age-related spatial processing. Classical accounts emphasize right-hemispheric dominance for visuospatial attention, whereas the HAROLD model proposes reduced hemispheric asymmetry and greater bilateral recruitment in older adults (Cabeza 2002). Recent work on hemispheric asymmetry and three-dimensional spatial processing suggests that age-related changes in spatial attention may influence how near and far space are represented (Britt et al. 2026). The present behavioral data cannot distinguish between right-hemispheric dominance and compensatory bilateral recruitment, because the study did not include lateralized spatial-attention tasks, line-bisection measures, or neurophysiological recordings. Future studies should therefore combine blindfolded tool use paradigms with lateralized spatial-attention tasks and neurophysiological measures to test whether age-related changes in spatial updating contribute to altered tool embodiment.

### Limitations and future directions

Several limitations should be considered. First, the older adult group was smaller than the younger adult group, which reduced sensitivity to small effects and especially to higher-order interactions. Sensitivity analyses indicated that the study was mainly powered to detect medium-to-large or large effects. Accordingly, non-significant small effects, particularly age-related moderation effects, should be interpreted cautiously rather than as evidence for equivalence. Second, training duration differed between age groups for safety reasons. Younger adults completed longer training blocks, whereas older adults completed shorter blocks to reduce fatigue, discomfort, dizziness, and fall risk during blindfolded navigation. Consequently, age-related differences may partly reflect differences in tool use exposure rather than age alone. Future studies should use larger and more balanced samples and should match training duration across age groups where safety permits, or statistically model exposure duration as a continuous predictor. Third, the study did not include a sighted control condition. Therefore, the present findings cannot determine whether visual deprivation reduced ownership, agency, forearm tactile distance perception, or RDE relative to normal vision. Future studies should include both blindfolded and sighted tool use conditions within the same design to directly test the contribution of visual feedback. Fourth, the RDE task was used as an explicit proxy for perceived reachability and action-scaled spatial representation which is in line with recent studies (Jahanian Najafabadi et al. 2026a, b a, 2026 b). This measure should not be equated with all forms of peripersonal space, including defensive or multisensory-facilitation peripersonal space (Zanini et al. 2021). Future work should combine RDE with independent measures of multisensory peripersonal space, such as audio-tactile or visuo-tactile interaction tasks, to determine whether reachability updating and multisensory peripersonal space modulation dissociate after blindfolded tool use. Fifth, no formal cognitive screening measure, such as the MoCA or MMSE, was administered (Folstein et al. 1975; Nasreddine et al. 2005). Although participants reported no known neurological or neuropsychiatric disorders and were able to understand the task instructions, subtle cognitive differences within the older-adult group cannot be ruled out. Future studies should include standardized cognitive screening to better separate age-related changes in tool embodiment from possible effects of cognitive variability. Sixth, participants reported no prior cane-use experience and were fully mobile. However, future research could compare participants with and without prior experience using mobility aids or similar tools. Previous experience with mobility aids or similar tools could influence tool use strategies, confidence, agency, ownership, and behavioral recalibration.

Another limitation is that the present design did not independently measure baseline attentional allocation across egocentric depth. Previous work suggests that perceptual resolution and spatial attention can differ between near and far space, with near-space advantages reported in several visual-attention and perceptual tasks (Ahsan et al. 2022; Blini et al. 2018; Britt et al. 2025; Britt and Sun 2024; Previc 1998). Therefore, target-search performance and reaching distance estimates in the present study may partly reflect depth-dependent attentional or perceptual gradients rather than tool-related recalibration alone. This issue may be particularly relevant when comparing younger and older adults, because age-related changes in spatial attention could alter how near and far space are sampled during blindfolded tool use. Future studies should combine tool use paradigms with independent measures of near/far spatial attention, such as depth-based target detection, localization, or line-bisection tasks, to determine whether tool-related changes in reachability estimates can be separated from baseline attentional biases along the longitudinal axis.

Finally, the present study used explicit agency ratings and did not include an implicit agency measure, such as intentional or temporal binding. Therefore, we cannot determine whether the age-related patterns observed here reflect changes in motor intention, action–effect prediction, causal inference, or response-level interpretation of agency ratings. Future studies should combine blindfolded tool-use training with implicit agency measures to test how motor intention and action-effect binding contribute to tool-related agency across age groups.

The present findings may also have preliminary implications for assistive-tool training and rehabilitation. The preservation of agency under blindfolded cane use suggests that active cane training can support subjective control over the tool even when visual information is unavailable. However, the weak ownership ratings indicate that short-term blindfolded practice alone may be insufficient to produce a strong conscious feeling that the cane belongs to, or becomes part of, the body. Future intervention studies could test whether longer training, repeated sessions, gradual integration of visual feedback, or enriched multisensory feedback strengthens both agency and ownership. Such work would be relevant for older adults and for individuals who rely on assistive mobility tools in daily life, where effective tool use may depend not only on motor control but also on confidence, safety, and flexible updating of action-related body representations.

## Conclusion

The present study shows that short-term blindfolded cane use elicits stronger explicit agency than ownership in both younger and older adults. This pattern should not be interpreted as evidence that visual deprivation selectively impairs ownership, because no sighted control condition was included. Rather, the findings indicate that participants can experience control over a voluntarily moved cane without necessarily experiencing the cane as part of the body. Forearm tactile distance perception and perceived reaching distance were more consistently associated with agency than ownership, especially during navigation. In the participant-level joint analyses, agency during navigation was the clearest outcome explained by the model, with significant contributions of perceived reaching distance. Age-related interpretations should therefore remain cautious because the older adult sample was smaller, training exposure was shorter for safety reasons, and the study was mainly sensitive to medium-to-large or large effects. Overall, the findings suggest that blindfolded cane use can support subjective agency and action-related recalibration, whereas explicit ownership over the cane remains weak after short-term training.

## Data Availability

Datasets led to the presented results can be accessed upon request and will be made available through OSF upon publication through https://doi.org/10.17605/OSF.IO/Y5J2Z.
